# Removal of the Uterine Appendages

**Published:** 1886-03

**Authors:** J. Greig Smith

**Affiliations:** Surgeon to the Bristol Royal Infirmary


THE BRISTOL

Medico-Chirurgical Journal.

MARCH, 1886.

### REMOVAL OF THE UTERINE APPENDAGES.

BY

J. GREIG SMITH,

*Surgeon to the Bristol Royal Infirmary.*

*Nomenclature.*—The want of a good name for this operation is already being felt. At first, when its object was supposed to be limited to the production of an artificial menopause, the operation was known as "normal-ovariotomy." Battey, who introduced the name, was among the first to recognise its unsuitability. As a matter of principle, the operation was by no means restricted to ovaries that were normal; and, as a matter of practice, it was found that most of the ovaries removed were actually diseased. The term, "Battey's operation," while suitable within the limits which Battey laid down for it, does not include the more extended modern proceedings. "Oöphorectomy" had already been used by Peaslee and others as a synonym for ovariotomy before it was sought to limit it to removal of small ovaries; and as oviducts are now, in most cases, removed as well as ovaries, the term is in a double sense objectionable. In connection specially with disease of the Fallopian tubes, Tait's name became associated with removal of the uterine appendages; and when, in harmony with certain beliefs which he holds as to the functions of the oviducts, he practised removal of the tubes as well as of the ovaries where others had been content with removal of the ovaries alone, the proceeding of "Removal of the Uterine Appendages" came to be known as Tait's operation. Men performed Tait's operation with Battey's motives; hence a confusion which has been rendered more confounded by more than one surgeon calling it the "Battey-Tait operation." The German terms, "spaying" and "castration," are objectionable on the grounds both of good taste and of exact naming. In many of the operations performed the effect of castration, as usually understood, is an undesirable accident rather than a definite aim. "Salpingectomy" for removal of the tubes, and "salpingo-oöphorectomy" for removal of the appendages, are fairly exact but decidedly cumbrous. "Prosthekotomy" is as applicable to caudal as to uterine appendages. A classical friend has suggested to me the word "thelytectomy" (Θηλύτης=feminine principle); but this, perhaps, is too suggestive of castration. Under any of these names, it is impossible to give a complete account of any recognised surgical operation; therefore, in place of a better, the vague but comprehensive title, "Removal of the Uterine Appendages," is adopted. Even to this name the objection may justly be raised, that the uterus is an appendage to the ovaries, rather than the ovaries to the uterus. From a surgical standpoint, however, the objection has less weight than from a physiological.

*History.*—As a barbarous custom the castration of women dates from a very remote antiquity. In the case of female domestic animals, such as cows, camels, sows, bitches, mares, and ewes, we have abundant evidence in the writings of Aristotle, Pliny, Galen, Suidas and others, that castration was very generally practised. The practice is said to have been extended to women at the instigation of certain kings of Lydia. According to Xanthus, a Lydian historian who wrote in the sixth century before Christ, the Lydian king Andramyte first introduced female eunuchs into the service of his palace. Gyges, another king of Lydia, is said to have caused the removal of ovaries from women with a view to prolonging their charms—"*quo illis semper aetate florentibus uteretur.*" Various authors have thrown doubt on the reality of this proceeding, saying that the so-called castration was either removal of the uterus (which is most improbable), or ablation of the nymphæ or the clitoris, or even (which seems an unwarrantable postulate) padlocking or "infibulation," a proceeding analogous to that which Celsus describes as having been carried out on boys. If the Lydians really castrated animals for domestic purposes,—and of this there can scarcely be a doubt,—it is quite within the bounds of possibility that they castrated women also. It is certainly unlikely that with the Lydians any other form of mutilation than removal of the ovaries should be considered as constituting castration.*

* Many classical writers of the Middle Ages refer to the practice. Foi more detailed accounts see—

Dujardin, *Hist. de la Chirurgie.* Paris, 1774.

Mahon, *Médecine Légale et Police Médicale.* Paris, 1801.

Much interesting information will be found in the second volume of Pierre Dufour's laborious work, *Histoire de la prostitutim chez tous les peuples du monde;* and a moderately full summary, with numerous references, is given by Boinet in the article, "Ovariotomie," in the *Dict. Encycl. des Sc. Méd.*

Even in times comparatively modern it is stated that the practice has been in vogue in Central Asia. A medical missionary, Dr. Roberts, is said to have met, in Bombay, Hedgeras — attendants in harems — who were spayed.* He remarks that they did not menstruate, and that they had no sexual desire.

The story of the Hungarian sow-gelder who, in a fit of parental anger, castrated his unchaste daughter, is supported by testimony of real weight.† Schurigius speaks of two similar cases; and other cases, with less weight of authority, have been recorded.

These ancient practices were not, however, therapeutic measures. Among the earliest operations performed with a beneficent purpose is that related by Franck de Franckenau, in which an ovary, prolapsed through a wound accidentally made over the pubes, was successfully removed. Percival Pott's well-known case of removal of herniated ovaries occurred in 1756. Thereafter the history of the operation is merged in that of modern ovariotomy for tumours.

In 1823 James Blundell, of Guy's Hospital, a man far in advance of his times, suggested, in a paper read before the Medico-Chirurgical Society of London, removal of healthy ovaries in cases of severe dysmenorrhœa, or metrorrhagia from inverted uterus. His advocacy of removal of the whole uterus in place of ordinary Cæsarean section, and the scientific manner in which he sought to sustain his arguments by experiments on rabbits, have scarcely received the attention which they deserve. As more germane to the subject in hand, special reference must be made to his proposed method for producing sterility in

* "Hedgeras de l'Asie Centrale," *Journal l'Experience*. 1843.

† See particularly Wierus, *Opera*, lib. iv., cap. xx.

cases of malformed pelvis. His words are as follows: "I would advise an incision of an inch in length in the linea alba above the symphysis pubis; I would advise, further, that the Fallopian tube on either side should be drawn up to this aperture; and, lastly, I should advise that a portion of the tube should be removed, an operation easily performed, when the woman would for ever after be sterile."*

Dr. Robert Battey, of Rome, Georgia, generally gets the credit of having been the originator of the operation as it stands in modern surgery. In 1865 he "conceived the idea of producing an artificial menopause for the remedy of disease;" but he did not publish his ideas till 1872. In February, 1872, Lawson Tait removed with complete success an ovary the size of a pigeon's egg, which contained a chronic abscess. This he claims† as being "the first record in the history of surgery of the removal of a small ovary for pain." Encouraged by this success, on August 1st, 1872, he successfully removed both ovaries for the purpose of arresting intractable hæmorrhage. (By some mistake Battey records this case as being fatal.) A few days before the date of Tait's second operation, Hegar operated on his first case with fatal result. On August 17th, 1872, Battey performed his first operation, which was successful, and in the following month he published it.‡

In discussing the history of the operation, it may be objected that Tait's first operation was not performed with the aim which Battey proposed. The ovary was felt enlarged in Douglas' pouch, and as only one ovary was removed, the operation was not intended to "produce

* *Principles and Practice of Obstetricy.* Ed. by Th. Castle. Lond., 1834. P. 580.

† *Dis. of Ovaries.* 4th Ed. P. 324.

‡ *Atlanta Med. and Surg. Journal,* Sept., 1872, p. 321.

an artificial menopause." There is no suggestion that the proceeding was the fruit of any preconceived theory: it was a local operation for a local disease. But who shall say of the original work of a practical man that it was not preceded by theory? Tait's second case was undoubtedly a physiological and pathological experiment; in favour of its probable success only theoretical reasons could be advanced. Hegar, too, must have independently thought out the operation; it has certainly been his fortune materially to influence its progress. To claim a priority measured by days or months is to be exact at the expense of liberality or even of justice; the time was ripe for the operation, and three independent workers—Battey, Tait, and Hegar—may be permitted to share the honour of introducing it.

As might be expected, the work of these three men has tended to run in different directions. Battey's name has continued to be associated chiefly with the operation as performed for what may loosely be called "neuroses." Tait's name is mainly connected with inflammatory diseases of the tubes, and his influence has been strongly felt in the substitution of operation for actual disease as against vague nerve-symptoms. Hegar, again, is best known in connection with the operation for uterine myoma. Trenholme, in January, 1876, would seem to have been actually the first to remove ovaries for bleeding myoma; but his influence has not been so great as that of others.

The practice of numerous followers in the footsteps of these pioneers has widened the range of the operation, at the same time that it has narrowed down its indications. Greater exactitude in diagnosis, and increasing knowledge in pathology, have largely replaced functional neuroses by palpable disease. Except for uterine myoma, the field for spaying, properly so-called, is being narrowed; on the other hand, the field for the removal of incurably diseased organs is being immensely widened.

*The Aim of the Operation.*—The purpose of the operation, as enunciated by Battey, was "to determine the change of life for any grave disease which is incurable without it, and which is curable with it." Though this definition may have held good for his own operation in its first conception, it is obviously imperfect as regards the operation in its modern development. In fact, a complete change of front would probably cover more cases: it would probably be more correct to say that removal of the uterine appendages is performed for local disease in the ovaries or tubes, than that it is done "to determine the change of life." But the operation has more than one aim; the definition of its purpose cannot be gathered into one sentence. It has a threefold purpose: (1) to remove organs incurably diseased; (2) to check or modify the discharge of blood from the uterus; and (3) to completely abrogate the process of ovulation.

The removal of organs incurably diseased, and causing danger to life or serious disablement, is, undoubtedly, the most justifiable aim of the operation. Under this head we have to deal with such conditions as abscess in the ovaries or tubes, Fallopian pregnancy, and strangulated ovarian hernia which cannot be reduced; these endanger life: also with the various cystic diseases of the tubes, the more chronic and subacute inflammations of the ovaries, and their displacements; these cause disablement in varying degrees.

It may be necessary to check bleeding from the uterus, either on account of its being excessive in amount, or because its discharge is attended with danger or great pain. Uterine myoma is the chief cause of metrorrhagia; incurable obstruction in the vagina or elsewhere to the menstrual flow causes both pain and danger; and certain malformations and malpositions of the uterus may be attended with so much pain at menstrual periods as to render life a sort of recurrent martyrdom.

To unsex the woman—the aim sought when castration is spoken of—is the least definite and the least satisfactory purpose of the operation. The ill-defined class of so-called reflex neuroses is not here specially meant: all, or at least the great majority of, inflammatory diseases of the appendages are attended by "reflex neuroses." Here we particularly refer to actual nerve diseases, such as mania or epilepsy, which we have reason to believe are either caused or kept up by the processes attendant on ovulation.

These theoretical "aims for operation" will be put into actual concrete and positive "indications" when the diseased conditions are specified and described. But with the purpose sought we must reckon the result achieved; and this result often oversteps the purpose. We may desire to put a stop only to that part of the sexual process which consists in the discharge of blood from the uterus, whereas the actual effect of operation is to strike at the root of the whole function of ovulation and destroy it. What exactly is the value of this *per contra* we have to reckon with?

Here we have to deal with sentiment as well as with science. The question is dragged hither and thither between the practical enthusiasm of the operative surgeon and the destructive criticism of the arm-chair theorist. The former presents his holocaust of ovaries removed, with all the pride and some of the contempt of the warrior; the latter rhapsodises on the sanctity of the sexual function, and seeks to inaugurate a sort of modern Phallic worship applied to the ovaries. It is the pride and glory of abdominal surgery that it lives and thrives upon statistics; and it is, perhaps, true that some men ask us to estimate their capacity in general by their experience in detail. This is a fault, but it is a fault in the right direction. And here the legal maxim, "*Ex abusu non arguitur in usum*," holds true. The evils produced by some men doing too much will never be counterbalanced by other men doing too little.

Practical men care little for fine-spun theories; they want to get their patients well. If objection is taken to the operation on the ground of the loss of sexual feeling, they say that this is a petty and contemptible thing to be weighed against prolonged suffering. If loss of the power of reproduction is the complaint, this is a weighty reason,—one in which those first interested must have the last word. Put to the patient herself, or to her husband, these objections—if the case is one in which operation ought ever to be contemplated—are usually promptly and summarily dismissed. And when the actual fact—that the womanly attributes are lost no more after the artificial than after the natural menopause—is borne in mind, the objections have still less weight. In most cases, child-bearing was either impossible or dangerous: here there is no loss; and, in many, dyspareunia is changed into eupareunia: here there is a gain. The general effects of the operation are nowhere better expressed than by Köberlé, as translated by Barnes:* "The subjects may be regarded as women who have suddenly attained the menopause. The affective sentiments remain untouched. They are no longer under the dominion of an

* "On Hernia of the Ovary," *Am. Journ. Obstet.*, January, 1883, p. 22.

imperious erotic want; but they are not the less good, loving towards relatives and husband. The genital organs remain excitable; the character becomes gentler, less irascible; the breasts do not atrophy; the tone and voice are unaltered." In one sentence, the change is one from active, excitable uxoriousness to staid, gentle matronliness. There is nothing very repulsive in this. Here, so far, the matter may rest. The definite results for the definite diseases will be indicated further on.

Considerations that ought to have at least as much weight as the ethical or sentimental ones just mentioned are, firstly, the danger of the operation itself, and, secondly, the not absolute certainty as to permanent cure among cases that recover. The average operator cannot count upon a mortality of less than 8 per cent.; and, as results go, he may expect a perfect cure in no more than 90 per cent. of all the cases that recover from the operation. These are grave facts to be dealt with in an operation not always intended to save life. The patient may be suffering a prolonged martyrdom; but a surgical operation which does not bring cure is scarcely a respite, and death is a terrible penalty to pay for relief. On these grounds we ought to be assured, in the first place, that there is a clear case for operation; and, in the second place, that the full ratio of probability of favourable result belongs to the case under consideration.

*Conditions indicating Operation.*—The disease—the extent of it and the symptoms which it produces—is the final criterion as to operative interference. It serves no good scientific purpose to describe a symptom as a cause of operation; it is unfortunate that so many operations are recorded as being for ovaralgia, dysmenorrhœa and such-like. The evil has not been diminished by the recent appearance of certain valuable papers in German periodicals, entitled "Castration for Neurosis." In most of the cases described pain was the only neurosis, and in most of them also there was actual disease in the appendages. It is just as scientific to speak of excision of the hip for reflex pain in the knee, as of excision of the appendages for reflex pain in the back. Wherever it is possible the disease ought to be quoted as the cause for operation, and not the symptoms of it. In a small and diminishing class of cases a profound neurosis, mania or epilepsy, may, under very special restrictions, be quoted as indications to operation. Yet even in these, with a curious frequency, disease of the organs is found at operation. For the rest, the disease will be spoken of as the indication for operation. Thus, instead of speaking of removal of the appendages for ovaralgia, dysmenorrhœa, or menorrhagia, we speak of the operation as being for ovaritis, pyosalpinx, or myoma. With this view the indications for operation may in skeleton outline be presented as follows:

A—The Appendages.

(1) *The Ovaries.*

(*a*) Inflammation—acute, chronic and suppurative (abscess).

(*b*) Displacement (prolapse, hernia).

(*c*) Cirrhotic and cystic ovaries.

(2) *The Fallopian Tubes.*

(*a*) Inflammation—Salpingitis.

(*b*) Pyosalpinx.

(*c*) Hæmatosalpinx.

(*d*) Hydrosalpinx.

(*e*) Fallopian pregnancy.

B—The Uterus.

(*a*) Uterine myoma.

(*b*) Errors of development—absence or mal-development of uterus with menstrual molimen.

(*c*) Incurable displacements with severe nerve symptoms.

(*d*) Insuperable obstruction to menstrual flow (may reside in vagina).

C—The Nervous System.

(*a*) Mania; puerperal mania, menstro-mania, nympho-mania, &c.

(*b*) Epilepsy; hystero-epilepsy, convulsions, cramp, dancing fits, &c.

(*c*) Hysteria.

It need scarcely be pointed out that the mere existence of any of these abnormal conditions is (with possible exceptions in the case of three or four of them) no indication for operation. The essential concomitants of these diseases must be of a grave nature—there must be danger of life or serious impairment of health—before operation is contemplated. With this preliminary and comprehensive proviso we may proceed to consider the indications in detail.

*Ovaritis, Oöphoritis, Inflammation of the Ovaries.*—The chronic form of ovaritis arising from excessive functional activity, which is found in prostitutes, rarely calls for operation. So also those temporary acute congestions, as obscure in origin as they are in pathology, require no notice. The majority of cases requiring operation have their origin in gonorrhœa. Septic matter may be carried along the tubes to the ovaries by puerperal inflammation in the uterus, and there is no doubt that a septic catarrh of the endometrium set up by traumatism, as from the sound or a tangle tent, may also be a cause. The exanthematic fevers and acute rheumatism sometimes beget a form of ovaritis which is attended with troublesome symptoms. Cases following septic inflammations at childbirth always pursue a rapid course and eventuate in abscess, which may be rapidly or even suddenly fatal, or may pursue a more chronic course, bursting into one or other of the neighbouring hollow organs. Following gonorrhœa, or simple traumatism, or any of the exanthemata, ovaritis is liable to become very chronic, and here indications to operate are most frequent and most legitimate.

In such cases the local signs may be marked enough, but urgency is usually bespoken by the reflex or the functional symptoms. The ovary is exquisitely tender to all mechanical disturbance, when the patient stands or moves quickly, and it drags on its ligaments or is jerked; and when it is pressed upon by the fingers through vagina or parietes. Engorgement at the menstrual period aggravates the pain. In the intervals also it causes pain, local on various provocations, and outlying and reflex in the groins, in the back, down the thigh, in the hypochondrium, and elsewhere. Almost every known form of functional sexual derangement may be associated with ovaritis,—dysmenorrhœa, menorrhagia, amenorrhœa.

Some weight must be given to the physical signs. An inflamed ovary has a tendency to fall downwards into Douglas' pouch and to become fixed there. Here it may be felt exquisitely tender, and sometimes causing a peculiar feeling of nausea on being pressed upon. In this situation it may readily be mistaken for the fundus of a retroverted uterus. A skilled diagnostician will at once recognise the shape and consistency of the ovary; he may even palpate its ligaments, and with a high degree of probability diagnose the presence of fluid or cysts in its substance. In thin patients the unprolapsed ovary may be palpated between the fingers in the vagina and on the parietes.

In a case diagnosed as chronic ovaritis, from whatever cause arising, if remedial treatment has had a full and fair trial, and if the patient's health is being undermined by the constant pain and other accompaniments, operation may be recommended. If abscess exists, life is endangered and operation is imperative. In the former case the indication follows on the gravity of the symptoms; in the latter the indication is positive and absolute on the existence of the disease.

*Displacements of the Ovary—Hernia: Prolapse.*—Among the first, if not actually the first, oöphorectomies for a beneficent purpose were one for *hernia of the ovaries* through a wound, and one for hernia into the inguinal canal. Hernia in itself is not an indication for operation. The herniated organs must be irreducible or the source of great pain, or the seat of some form of degenerative or inflammatory disease, before operation is contemplated. A traumatic hernia following strain or parturition is not so likely to be attended with troublesome symptoms as a congenital hernia. The latter variety is twice as frequent as the former, and is usually not of so simple a nature. Congenital hernia always contains tube as well as ovary; not infrequently it is associated with uterus bicornis, and one horn of the uterus occasionally follows the appendages into the hernial sac; also under-developed uterus, with its accompanying symptoms of aggravated molimina, may complicate the condition. In at least six cases complete absence of the uterus has been noted as accompanying inguinal ovarian hernia.

The diagnosis is not often difficult. The characteristic size and shape, the sensations upon pressure, the increase in size at the menstrual periods, and the frequent association with malformations of the uterus, form a combination which can scarcely be imitated by any other condition. A sign of importance, where it can be elicited, is an associated movement with lateral displacement of the uterus.

The tumour increases in size at the periods, and becomes more tender. Then the symptoms may mimic those of strangulated hernia. Actual strangulation may take place, and then operation is imperative. So also if extra-uterine pregnancy takes place in the sac, an occurrence of curious frequency, operation is indicated. And any degenerative or novel development in its tissue, cystic or glandular, demands operation. Otherwise the indications rest upon its resistance to palliative measures, its irreducibility, and the urgency of the symptoms which it produces.

*Prolapse of the ovaries* is a condition by no means always demanding surgical interference; it is often accidentally discovered during examinations for other purposes. Symptoms of urgency are produced only when they become inflamed or are bound down by adhesions. The existence of adhesions between mobile abdominal organs as a cause of pain, or other abnormal symptoms which may be of grave import, has probably not received the attention which it deserves; in the case of the ovary, from the nature of the organ, it is certain that this condition is peculiarly liable to breed trouble. Adhesions in

Douglas' pouch are most common, but they may be attached to almost any part of the intestinal or pelvic peritoneum. I have successfully operated upon a case where the left ovary was closely adherent on one side to the sigmoid flexure and on the other to the tip of the vermiform appendix. The ovary being dragged upon between these restless organs, it was not surprising that acute symptoms of ovarian irritation were produced. Sometimes mere prolapse started by congestion may be continued by a sort of strangulation of the blood supply; in such cases operation may be required, though no adhesions exist. Oöphoraphy, or fixation of the ovary by a special proceeding, has been devised to meet this condition; the value of it has yet to be decided. The left ovary is more frequently prolapsed than the right, probably because it is more liable to become congested on account of the left ovarian vein having no valve.

*Cystic and Cirrhotic Ovaries.*—Though it is probable that small cystic ovaries have their origin in chronic inflammation, and it is almost certain that cirrhosis or fibroid thickening of the ovaries claims this cause, yet in actual practice the condition is not found to be inflammatory. In the cystic ovary the disease is not that of ordinary cystoma, a glandular new growth, but simply a distension of follicles in the thickened and contracted stroma. In true cirrhosis the gland tissue is replaced, in whole or in great part, by fibrous material; the surface is puckered and scarred, and the size of the organ is diminished. It would seem that one termination of the ovaritis found after the exanthemata, especially after scarlet fever, is in this cirrhotic atrophy with follicular distension. The pathology of this condition is still but ill understood.

It is a clinical fact that small cystic ovaries are often attended with profuse and uncontrollable menorrhagia. On the other hand, pure cirrhotic atrophy may be accompanied by amenorrhœa, though the molimina may be excessively severe. In many all the varied and shifting symptoms of deranged sexual function are present in their most aggravated forms. And in such—partly, no doubt, because local disease cannot be physically made out—ovarian neuroses are abundantly called in to justify operation. Taking Schmalfuss's statistics of Hegar's operations as fairly representative, quite one-half of the so-called functional neuroses own such parenchymatous changes in the ovaries as causes.

These cases run a very prolonged course; they are peculiarly resistant to palliative measures, and the reflex disturbances are liable to be marked and numerous. If the patient cannot put up with her troubles, and the menopause is not near, operation must be performed: it is the only cure.

*Diseases of the Fallopian Tubes.*—Half a century ago, diseases of the Fallopian tubes were well described by more than one writer; but so completely had all knowledge of them dropped out of mind, that when, a little while ago, a distinguished provincial surgeon published results of operations for such diseases, an equally distinguished metropolitan brother expressed a somewhat emphatic disbelief, not only in the operation, but in the diseases as well. It betokened an extraordinary, and almost discreditable, ignorance of the work of our predecessors that Tait found it necessary to send far and near specimens of these diseases, in order to establish a belief in their reality. It is well enough known now that diseases of the oviducts are a prolific cause of serious functional disturbances, and that they may even cause death. The difficulties are now not so much theoretical as practical: how to diagnose them, and how best to treat them; when palliative measures may be persevered in, and when operation becomes necessary.

Clinically, it is impossible to separate simple salpingitis from hæmatosalpinx and pyosalpinx. The last two may, in fact, be considered as varieties of the first. When the septic inflammation reaches the fimbriæ, it binds them together and to the ovaries, sealing up the opening. The opening into the uterus is either closed or too small to permit exit to all the fluid; thus we have catarrhal inflammation in a closed sac, the mucous lining of which bleeds every month. While the peritoneal covering exhibits the usual changes consequent on inflammations of serous surfaces, the cavity may contain blood or pus, or a mixture of both. The amount of the blood or pus contained in the tube varies greatly. Sometimes it may be less than a drachm: collections of blood rarely exceed an ounce. I have removed a Fallopian abscess which contained more than a pint of putrid pus along with a considerable amount of gas.

Most cases of salpingitis are septic, the result of inflammations of the endometrium, gonorrhœal or puerperal. Some cases originate in leucorrhœa, and in such Wylie tells us he has, by squeezing the tubes, made the pus flow into the uterus and appear in the vagina. A few are caused by syphilis; some are tubercular.

An inflamed and engorged or distended tube usually falls downwards into Douglas' pouch, and becomes more or less intimately adherent there. By the vagina it may be felt as a moderately soft, boggy, irregularly-rounded tumour, not unlike the fundus of a retroverted uterus. It is exquisitely tender to the touch, causing dyspareunia, and cannot readily be pushed upwards. Attacks of pelvic pain, greatly increased at the periods, but aggravated at other times with or without provocation, accompany the complaint. Menstruation is fitful and irregular, usually increased, but sometimes diminished in flow. Each period, in hæmatosalpinx, adds to the danger as well as to the pain. Most cases occur in married women, and, according to Tait (to whom, more than to all others, we are indebted for our clinical knowledge of the disease), "a very frequent feature in the history of the cases was found to be that they had one child, and after that were never free from pain till relieved by the operation."

In every case of true and persistent salpingitis operation is indicated. With judicious treatment and rest, mild cases may get well; but when the ends of the tubes are blocked, and they contain pus or blood, removal gives the only chance of relief. It must not be forgotten, as an indication to operate, that there is real danger to life in septic inflammations of the Fallopian tubes.

Hydrosalpinx is a milder affair, and may be attended with very few symptoms. In many cases it is simply a retention cyst in a functionally inert duct containing clear fluid; in others, epithelial *débris* or pus, sometimes mixed with blood, is found. The cause of the occlusion being usually inflammation extending from the uterus, both tubes are often found blocked. In a case on which I operated, one tube contained several ounces of clear fluid; the other contained a fluid so thick with flakes of cholesterine that it looked like molten silver when poured from one vessel into another.

The diagnosis is physical. A sausage-shaped or tortuous cyst in the retro-uterine space, with symptoms of disturbed sexual functions, worst at the periods, and less severe than in acute salpingitis, suggest the disease.

As to operation, hydrosalpinx is said—I believe on insufficient grounds—sometimes to last a lifetime, producing no symptoms and causing no harm. In many cases the symptoms are so severe as to demand operation; in all operation is advisable. The disease will not improve; from accidental or other causes it may suppurate and burst, and so endanger life.

*Fallopian Pregnancy.*—As I cannot here enter into the whole question of extra-uterine pregnancy, any statement of belief as to the expediency of operative interference may seem bald and dogmatic. Most men are now agreed as to the truth of Tait's opinion, that all examples of extra-uterine gestation are in the beginning either wholly or partially Fallopian. The risks of rupture before the fourth month are very great; and danger to life has by no means passed when the ovum is killed by electricity or otherwise.

I hold strongly to the belief that as soon as an extra-uterine pregnancy has been diagnosed, operation ought to be performed. Though Thomas and others claim to have diagnosed the condition before rupture, it is unfortunately the case that the first sign of it usually appears at rupture, when the woman is dangerously ill from intra-abdominal bleeding and shock. In such an event it would be a surgical crime to let the patient die without an attempt at relief. But under all circumstances, the safety of the patient, immediate as well as remote, is best consulted by removing the ovum with the tube at the earliest possible period. According to Parry, the mortality of extra-uterine pregnancy is 67·2 per cent.; early operation in competent hands would certainly give a death-rate ten times less. In the hands of Tait, whose operations have all been at the time of rupture, the mortality has been even less than this. In America, where electricity is in vogue for these cases, the successes claimed rest on doubtful diagnoses, and even then they do not equal those after operation.

*Conditions mainly Uterine.*—Of these, the condition requiring operation most frequently is myoma, or uterine fibroid. The exact position of the operation for uterine myoma is not yet fixed. It has been much discussed and written about at meetings and in journals; and the general outcome of the discussion has been that cause for interference resides, not so much in the tumour itself, its size or situation, as upon the symptoms it produces.

*Uterine myoma* has many phases. We find it frequently in *post-mortem* examinations of patients who have died of something else, and in whom it produced slight or no symptoms during life. We find it of moderate size in young women, where it causes excessive metrorrhagia, completely or partially disabling them. And most frequently of all it is met with in women near the menopause, as a large slowly-growing mass, prolonging the periods and causing excessive flow, but not endangering life or causing much disablement. Lastly, we have a class in which the tumour goes on growing till it reaches dimensions so great as to interfere with the vital processes. The rapidly-growing œdematous myoma of Tait deserves special mention. These groupings may be modified and interchanged in numerous ways; they broadly represent the conditions as most frequently met with, and that is all.

It is impossible, without fully entering into the whole question of the treatment of uterine myoma, to discuss the pros and cons of removal of the uterine appendages for it. The treatment has its origin in a *vera causa*—the atrophy of such growths with the natural atrophy, at the menopause, of the reproductive glands. By removal of the central glands we seek to produce atrophy of the organs whose functions are dependent on these glands. The results have been encouraging, and the operation is received with growing favour. Even in the hands of such skilled hysterectomists as Keith, Thornton, and Bantock, removal of the appendages is a favourite operation. Tait has always been its chief advocate.

The indications for removal of the appendages cannot easily be laid down in general terms. The leading indications are hæmorrhage and rapidity of growth. Circumstances of weight are, the age and condition of the patient and the size of the tumour. In very large tumours it is not easy, and sometimes impossible, to remove the appendages. If the patient is near the menopause, we may temporise, using the ordinary remedial measures; if the patient is under thirty-five, we must take into account the past history of the growth as to rapidity of enlargement, and amount of hæmorrhage, and try to forecast the probable risks of leaving it. If the patient is married, the risks are increased from pregnancy. If she is in poor circumstances, forced to earn her living, operation may be indicated, when in a well-to-do patient, to whom chronic invalidism is not an unmitigated evil, it may not be indicated. With the above reservations, free bleeding, which cannot be controlled by ordinary means, is the leading indication for operative interference. If a growth of small size is growing rapidly, and producing symptoms of its presence other than bleeding, and if the woman is some way off the menopause, we may interfere.

The general mortality of the operation for myomata is somewhere near ten per cent. In the hands of individual operators it is, however, less. As to the results, we may, in every thirteen cases of recovery after operation, reckon upon complete cure—*i.e.*, shrinking of the tumour and menopause—in ten cases; improvement in two; and failure in only one. But these results, with increasing care and judgment in the selection of cases and the period of operation, will no doubt improve. If cases of uterine myoma are kept under close and constant observation, and treated by removal of the appendages sufficiently early, the field of hysterectomy for this disease will become greatly diminished.

Certain congenital *anomalies and defects in the uterus*, associated with a normal development of the appendages, may be attended with moliminal disturbances so severe as to justify operation. These are: complete absence of the uterus; embryonic, fœtal or infantile uterus; the so-called uterus pubescens. In these the menstrual functions subsidiary to well-developed tubes and ovaries cannot be properly discharged. There is, not retention, but a complete absence or imperfect establishment of the menstrual flow. The uterus is overburdened by its ovaries and tubes, so to speak, and at the periods of menstruation it gives no relief to their engorgement by bleeding. The moliminal pains in such cases are often very severe, confining the patient to bed, driving away sleep, and requiring the administration of opium or even chloroform. In the intervals there may be comparative ease; but frequently the pain spreads over these intervals as well, and the patient becomes broken down in health and glides into chronic invalidism. It is often possible in such cases to detect the ovaries enlarged, prolapsed and tender, and the tubes are sometimes abnormally large as well.

The operation is sometimes called for in *incurable displacements of the uterus* when the symptoms are severe and irremediable. Retroversion and retroflexion, where the uterus has become adherent in its abnormal position, is the most frequent condition. The uterus is enlarged and tender, free bleeding takes place at the periods, pessaries are either useless or unbearable, or both, and other local measures, after prolonged trial, prove ineffectual. The patient is a chronic invalid, probably confined to bed or couch, and there is no prospect of cure. In such an extreme case removal of the uterine appendages, causing atrophy of the uterus, affords the only chance of cure.

*Insuperable obstruction to the flow of the menstrual fluid* is sometimes an indication for removal of the appendages. In such cases there is extreme pain at the periods, and sometimes, according to Battey and others, even danger to life. Injuries during labour, such as extreme cicatricial contraction of the vagina, and destruction with occlusion of the lower part of the uterus, are the most frequent causes. Congenital imperforate uterus is another cause.

The operation in *mania* has an extremely limited range. The proposal of Goodell to remove the ovaries from all female lunatics who have abnormal sexual propensities cannot be regarded seriously, any more than we should regard castration under similar circumstances in the male. Certain cases of mania, in which the attacks come on solely or chiefly at the periods, and in which a sexual element strongly predominates, might, under very special restrictions, be properly treated by removal of the appendages. In puerperal mania, particularly if the disease has recurred after a second confinement, the operation is specially indicated. In this case it would be castration pure and simple. For nymphomania the position of the operation is less assured. The operation has been performed for confirmed masturbation; but for this purpose it does not appeal strongly to the sympathies of the surgeon. Indeed, as the operation may not remove sexual feelings, it may not cure the habit.

In true *epilepsy*, the operation has not been attended with very favourable results. A few cases of recovery are recorded, but the accounts of these do not leave it certain whether the disease was true epilepsy or not. It is not sufficient to justify operation that the fits are worse or more numerous at the menstrual periods; this is true in many examples of confirmed epilepsy. Before operation is thought of, there must be a very strong presumption that the disease had its origin in perturbed sexual function, and the disease must not be so far advanced as to preclude the probability of cure. Actual disease in the appendages is an indication for operation.

In *hystero-epilepsy* there is, undoubtedly, a fair and promising field for operation. But here, also, the mere existence of the disease does not indicate operation. Clear and definite evidence of its connection with the sexual functions must be forthcoming. If the first fit occurred at the first menstruation; if subsequent fits occurred exclusively at the periods, and if they became intermenstrual only after having existed over several years; if there is an aura starting from the region of the ovaries; if the fits do not increase in severity; if they are unaffected by bromide of potassium; and, finally and most important of all, if there is palpable disease of the appendages, we may consider the case as suitable for operation. The minor symptoms of hystero-epilepsy, such as muscular cramps and spasms and disturbances of sensation, are not of importance surgically. Of more importance are the general condition of the patient as to health, and her position in life—that is, whether she must earn her living or not. If the health is failing, and the patient is becoming a helpless invalid; or if she is absolutely precluded from supporting herself by her own exertions, and has no future but the workhouse, these we should rightly consider as additional indications.

In not a few of the cases where cure has followed operation, there has been very slight or no disease of the appendages; in others, uterine stenosis and incurable displacements are found. The amount of disease does not *pari passu* increase the urgency of operation. Just as many women whose sexual organs appear to be perfectly normal, and who bear children, suffer more at their periods than others who are sterile from palpable disease, so may a slight amount of disease in one woman produce hystero-epilepsy, where in another it would produce no symptoms whatever. The index of excitability to reflex neuroses varies in different constitutions. In a sliding scale downwards, it is easy to reach the condition of no local disease at all, and speak only of disturbed function; and, according to a good few reports, we must at present accept this functional disturbance as a real cause. But it is only provisionally so accepted; future investigations will probably displace it altogether.

For *hysteria* the operation has been performed, and with success. But operations of less severity than removal of the appendages have been successful in hysteria,—a small cut on the abdomen, for instance. For mere hysteria, even if accompanied by "convulsive attacks" or "dancing fits," few surgeons would care to remove the appendages. The attacks would have to be very troublesome indeed, and the case would have to be surrounded by every conceivable inducement to operate, before interference could be contemplated.

*(To be continued.)*

